# Supplementary material for: The effectiveness of interactive mobile health technologies in improving antenatal care service utilization in Dodoma region, Tanzania: A quasi—Experimental study
Source: PLOS Digit Health. 2023 Aug 16;2(8):e0000321. doi: 10.1371/journal.pdig.0000321 (PMC10431653; doi:10.1371/journal.pdig.0000321)
Supplement: S1 CONSORT Flow Diagram — (DOC) [file pdig.0000321.s002.doc]

**
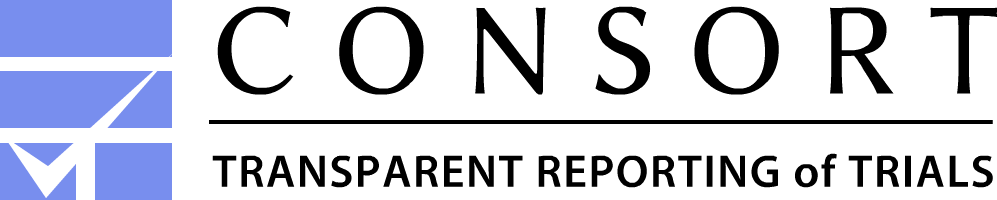
**

**CONSORT 2010 Flow Diagram**

**Allocation**

**Analysis**

**Follow-Up**

**Enrollment**

Assessed for eligibility (n=800) )

Excluded (n= 350 )

♦  Not meeting inclusion criteria (n=318 )

♦  Declined to participate (n=32 )

♦  Other reasons (n=0 )

Analysed (n=150 )

Lost to follow-up (give reasons) (n= 0 )

Discontinued intervention (give reasons) (n=0 )

Allocated to intervention (n= 150 )

Lost to follow-up (give reasons) (n= 0 )

Discontinued intervention (give reasons) (n=0 )

Allocated to control (n= 300 )

Analysed (n= 300 )

Randomized (n= 450)
