# Supplementary material for: The effectiveness of interactive mobile health technologies in improving antenatal care service utilization in Dodoma region, Tanzania: A quasi—Experimental study
Source: PLOS Digit Health. 2023 Aug 16;2(8):e0000321. doi: 10.1371/journal.pdig.0000321 (PMC10431653; doi:10.1371/journal.pdig.0000321)
Supplement: S1 File — (PDF) [file pdig.0000321.s003.pdf]

THE UNIVERSITY OF DODOMA

OFFICE OF THE DEPUTY VICE CHANCELLOR - ARC

DIRECTORATE OF RESEARCH AND PUBLICATIONS, CONSULTANCY AND INSTITUTIONAL COLLABORATION

P.O. BOX 259,
DODOMA, TANZANIA
TEL: +255 026 2310301 FAX: +255 0262310005 WEBSITE: www.udom.ac.tz

Ref: UDOM/DRP/134/VOL V/23-33 3rd April, 2018

To: Theresia J. Masoi
College of Health Sciences

RE: YOUR REQUEST FOR ETHICAL CLEARANCE

This is to certify that the proposal titled **"Testing the effectiveness of Interactive Messaging Alert System in Improving Knowledge on Obstetric Danger Signs, Service Utilization, Birth Preparedness and Complication Readiness Among Pregnant Women in Dodoma in Tanzania: A Quasi – Experimental Study 2018"** has been **granted** ethical clearance.

The Principal Investigator of the study must ensure that the following conditions are fulfilled:

1. Progress report is submitted to the University of Dodoma.
2. Permission to publish the results is obtained from the University of Dodoma.
3. Copies of final publications are made available to the University of Dodoma.

Approval is valid for a duration provided for under clause five (5) of the Ethical Clearance form.

Best Regards,

Prof. F. Fabian
Director of Research and Publication, Innovation, Consultancy and Institutional Collaboration

Cc: DVC - ARC
