# Supplementary material for: The effectiveness of interactive mobile health technologies in improving antenatal care service utilization in Dodoma region, Tanzania: A quasi—Experimental study
Source: PLOS Digit Health. 2023 Aug 16;2(8):e0000321. doi: 10.1371/journal.pdig.0000321 (PMC10431653; doi:10.1371/journal.pdig.0000321)
Supplement: S2 File — (DOCX) [file pdig.0000321.s004.docx]

Title: The effectiveness of interactive mobile health technologies in improving antenatal care service utilization in Dodoma region, Tanzania: a quasi - experimental study

AUTHORS:

Theresia J.Masoi1, Stephen M. Kibusi1, Deogratius Bintabara^3^*, Athanase G.Lilungulu^4^

^1^ Department of Clinical Nursing, the University of Dodoma P.O. Box 259 Dodoma, Tanzania

^2^ Department of Public Health and Community Nursing, the University of Dodoma P.O. Box 259 Dodoma, Tanzania

^3^ Department of Community Medicine, the University of Dodoma P.O. Box 259 Dodoma, Tanzania

^4^ Department of Obstetrics and Gynecology, the University of Dodoma P.O. Box 259 Dodoma, Tanzania

*Corresponding Author

Authors’ Email Addresses:

TJM: [jtheresia2008@yahoo.com](mailto:jtheresia2008@yahoo.com)

SMK: [skibusi@gmail.com](mailto:skibusi@gmail.com)

DB: [bintabaradeo@gmail.com](mailto:bintabaradeo@gmail.com)

AL: [athalilungulu@yahoo.com](mailto:athalilungulu@yahoo.com)

**Abstract**

**Background:** Unacceptable high maternal mortality rates remain a major challenge in many low income countries. In Tanzania, MMR per 100,000 live births was 578 in 2005, 454 in 2010, 432 in 2012. The most recent data in Tanzania shows further increase in MMR to 556 per 100,000 live births in 2015. The effectiveness of interactive mobile health technologies in improving antenatal care service utilization in Dodoma region, Tanzania: a quasi - experimental study

**Methods:** A quantitative quasi-experimental study with control will be carried. A purposeful selection of participants will be employed to achieve a sample size of 450 pregnant women (Intervention=150 and Control=300) who started their first antenatal visit below 20 weeks. Cases will be matched to controls by gravidity and gestational age at a ratio of 1:2.The intervention group are enrolled in an interactive messaging alert system and receive health education messages. The control group continues with the standard ANC services being offered in local clinics. A standardized semi-structure questionnaire adopted and modified will be used to collect data. Antenatal service utilization will be measured by using three indicators as it was used in i. ANC care provided by skilled health personnel (a nurse or a doctor);ii. Sufficient number of ANC visits (four or more visits during pregnancy); and iii. An appropriate ANC content provided (included at least 13 out of 15 of the recommended basic services/procedures or contents).Individuals scoring three of the basic components (attended by skilled health personnel, 4 or more visit during pregnancy with appropriate contents/services), will be classified as received adequate ANC, while those who did not comply with the three criteria will be termed as received inadequate care. Data will be analyzed by using SPSS software for both descriptive and inferential statistics.

**Discussion:** Interactive mobile health technologies have the potential to empower pregnant women through greater access to health information, and communications with their care providers, which may aid in self-care, shared decision making, early diagnosis and early care seeking behavior. it hoped that , the results of the study will be used by the country’s policy makers in developing appropriate policy that enhance utilization of all the recommended antenatal care services.

**Keywords:** Interactive messaging, Antenatal care, service utilization, Health education messages

**Background**

Health care services during pregnancy, childbirth and after delivery are important for the survival and well-being of both the mother and the newborn. Early detection and management of antenatal risk factors are critical for improving maternal and newborn outcomes. Timely and appropriate utilization of antenatal care can prevent complications and ensures better maternal and newborn health care [1]. The World Health Organization (WHO) recommends three core health sector strategies for reducing maternal and early neonatal deaths: comprehensive reproductive health care; skilled care for all pregnant women especially during delivery; and emergency obstetric care for all women and infants with life-threatening complications [2].

These strategies envision a world where every pregnant woman and newborn receive quality care throughout the pregnancy, childbirth and the postnatal period. Within the continuum of reproductive health care, antenatal care (ANC) provides a platform for important health-care functions, including health promotion, screening and diagnosis, and disease prevention [2]. ANC also provides the opportunity to communicate with and support women, families, and communities at a critical time in the course of a woman's Life [3] .

Developing countries carry the largest maternal mortality burden. According to WHO Maternal Mortality Fact Sheet (2016), the maternal mortality ratio in developing countries in 2015 was 239 per 100 000 live births versus 12 per 100 000 live births in developed countries (2). In Tanzania, the estimated MMR in the 2015-16 Tanzania Demographic and Health Survey TDHS-MIS was 556/100000 [ 4].

Women die as a result of complications during and following pregnancy and childbirth. Most of these complications develop during pregnancy and can be prevented or treated. Other complications may exist before pregnancy but are worsened during pregnancy, especially if not managed as part of the woman’s care. The major complications that account for nearly 75% of all maternal deaths are severe bleeding (mostly bleeding after childbirth), infections (usually after childbirth), high blood pressure during pregnancy (pre-eclampsia and eclampsia), and complications from delivery and unsafe abortion [5].

In this regard, Tanzania through the Ministry of Health Community Development Gender, Elderly and Children (MoHCDGEC) has adopted different strategies and efforts to promote safe motherhood and improve [child survival](https://en.wikipedia.org/wiki/Child_survival). Similarly, in an effort to improve maternal and child health, Tanzania’s government has declared maternal and child health services to be exempt from user fees in government facilities.

As part of the Sustainable Development Goals (SDG), the United Nations set a target to reduce the global maternal mortality ratio to less than 70 per 100 000 live births, between the year 2016 and 2030 with no individual country exceeding an MMR of 140 maternal deaths per 100 000 live births. As part of SDG number 3,Good Health and Well-Being for People, which aims to achieve universal health coverage to include access to essential medicines and vaccines [7] .

According to the TDHS report of 2015/2016, only twenty-four percent of pregnant women started antenatal care in their first trimester, and only one-half (51%) had four or more ANC visits as recommended. While in (2004/05) TDHS report, 94% of pregnant women made at least one antenatal care (ANC) visit and 62% of women have four or more ANC visits. The median number of months that women are pregnant at their first visit is 5.4 months [4] . Provision of advice during antenatal care about potential pregnancy complications and danger signs and information on how to seek medical care are viewed as key strategies to reduce delay in seeking skilled care [1].

Also as per TDHS2015/2016 report Sixty-three percent (66%) of births in Tanzania were delivered in health facilities and (66%) of mothers and newborns (58%) did not receive the recommended postnatal care within 2 days after birth [4]. Factors that prevent women from receiving or seeking care during pregnancy and childbirth include poverty, distance, lack of information, inadequate services and cultural practices [2].

Effective monitoring of pregnant women is essential for providing timely and necessary obstetric interventions as soon as problems arise. This study aim at testing the effectiveness of interactive mobile health technologies in improving antenatal care service utilization in Dodoma region in .

**Methods/Design**

**Study design**

The study is a quasi-experimental study design with control . The intervention group will beenrolled in the interactive messaging alert system and receive health education messages as per WHO guidelines, whereas the control group will receive the normal standard ANC services being offered in the local ANC clinics.

**Study area**

This study is carried out at Dodoma Municipal for both the intervention group and control group. Dodoma Municipality is found in Dodoma Urban District. Dodoma Region is one of Tanzania’s 30 administrative regions and the location of the capital city of the country. According to the 2012 Tanzania national census, Dodoma was one of the regions with the highest maternal mortality rates in Tanzania as it ranked the ninth high burdened region with a maternal mortality rate of 512/100,000 live births [8].Six health facilities within the Dodoma Municipal are involved, two being the interventions group and four for control groups

**Participants .** The intervention group will consist of pregnant women who began ANC care at less than 20 weeks gestation and will receive health education messages .The control group will consist of pregnant women who also began ANC care at less than 20 weeks gestation. However, this group will continues receiving the usual local facility ANC care; Controls will be matched to the intervention group by age group, education level, gravidity and gestational age.

**Inclusion and exclusion criteria**

All pregnant women who start their ANC first visit below 20 weeks, attending ANC and planning to deliver in Dodoma Municipal and who own phones and consert to participate will be considered for the study. Pregnant women who will be receiving pregnancy-related health text SMS from other sources through their mobile phones and those who meet the inclusion criteria and refused to participate will be excluded.

**Sample size**

The sample size for the Intervention and Control group will be obtained by using the following formula. Using formula for comparing two independent samples (Intervention group versus control group), and using proportion women attending four visit or more at baseline was 51% and after intervention it was 63% as per [9] , A Quasi-experiment that was done in Rural Uganda on Maternal Health Service utilization and Newborn care**.**

n =$2\{z\alpha\sqrt{\left[ \pi0\left( 1-\pi0 \right) \right]}+Z\beta\sqrt{\left[ \pi1\left( 1-\pi1 \right) \right]}\}/(\pi1-\pi0)2$ . Patra (2012)

n= Minimum sample size

Zα = Standard normal deviation of 1.96 at 95% confidence interval (CI)

Zβ = Standard normal deviation 0.84 with the power demonstrating statistically significant difference before (baseline) and after intervention between two groups at 90%.

π0= Proportion of an intervention group at baseline =51%

π1= Proportion after the intervention =63%

With 5% attrition rate.

n= $2\{1.96\sqrt{\left[ 0.51\left( 1-0.51 \right) \right]}+0.84\sqrt{\left[ 0.63\left( 1-0.63 \right) \right]}\}/(0.63-0.51)2$

n=2[(1.96×0.4999) + (0.84 × 0.4828)] /0.0144

n=2(0.9798+0.4056)/0.0144

n=2.7708/0.0144 =142

n=142

So, the minimum sample size was 142 plus 5% Attrition =150.

The ratio of the Intervention group to control was 1:2, so controls were 300.

So, the total sample size in this study was 450 pregnant women.

**Sampling technique**

A purposive sampling method will be used to get Dodoma Region, Dodoma Municipal and the Healthcare facilities offering ANC and delivery care services in Dodoma Municipal. Within both the intervention and control groups, participants will be selected by a systematic random method. In each setting, the criteria of less than 20 weeks gestation shall be utilized and attending ANC visit for the first time. Every third pregnant among those who meet the criteria and who will agree to participate in the study will be selected in both control and intervention group. The sampling frame will be Dodoma Urban District. Control and intervention group are from separate health facilities.

**Recruitment**

Seven research assistants who are medical personnel (doctor and nurses) are recruited and will be trained for two days on the objectives of the study, interviewing techniques and how to use the data collection tool in the field. The research assistants are all fluent in Swahili language. The principal investigator and the doctor are responsible in responding to the calls and text messages from the study participants.

**Intervention**

**Interactive messaging alert system**

The intervention aim at improving ANC service utilization . The intervention is implemented into two phases; The Intervention phase in which pregnant women in the intervention group are able to receive health education messages and call or text back to ask questions or clarification and the Post-intervention phase the questionnaire will be given to a women after delivery to see the changes to both the control and intervention group.

The application will be developed and moved to a server and connected to a mobile gateway with enhanced capability to handle multiple and simultaneous SMS problems from the system. Specially designed software automatically generated and sent text messages. The information required for the interactive messaging software such as gestational age and mobile phone number were gathered in the first visit and entered into system by the registering nurses with the help of the system administrator. The aim of the SMS components is to provide simple health education information. Text messages are sent as reminder to encourage routine ANC attendance and are sent to both expecting parents (mother and father). Providing SMS text messages to both partners was undertaken to influence male involvement in antenatal care service utilization. The content of the messages will be developed by an inter-professional team of nurse midwives and obstetricians from the College of Health Sciences at the University of Dodoma.

The communication is a two-way communication whereby participants are able to send and receive information about their health and pregnancy progress. With the interactive message, a pregnant woman could send text SMS through the system and reach a doctor or nurse. The health provider could respond the message through the system and it was directed back to the pregnant women. As such, one physician and the principal investigator, act as a triage helpdesk which allowed women registered on the system to ask maternal and child health related issues. If the message contained an urgent health matter needing specialist or urgent care, then the women was advised to visit or seek more information at the health facility. The health education SMS messages sent to pregnant women are free of charge for the recipients. The pregnant women are only charged if they call or text the system in which case, their cost was the standard charges by their individual network provider. Women were allowed to ask as many questions as they wanted and all the communication were recorded to the system database to identify the patterns of messages and the frequently asked questions.

### Quality checks up of the SMS

Message content will be checked for standard and provided as simple SMS in the local language of Swahili. The constructed SMS will not exceed 480 words equivalent to three SMS, sent at different interval of time depending on the gestational age. In the first trimester one message will besent per week, in the second trimester two messages per week and in the third trimester three messages per week. It will be done this way to avoid frequently repetition and irritations and to monitor the flow and frequencies of messages.

### Security, backup and recovering mechanism

The System is implemented with the backup mechanism. Whereby every day the system stored data to the backup device . This help in case of any crises data could be recovered on time. The system is also implemented with current technology to ensure security and prevent hackers to hack the system. The system is updated regularly to ensure any vulnerability/ open door is closed. There is a password that protect web user interface, and enabled every user to access only the information required at his /her position in the system. So every user was assigned an account. The System is now online as a pilot product (http://dodoma-antinetal-pns.or.tz).

Research instrument / Tool.

Semi-structured questionnaire (with both closed and open-ended questions) will be developed to be interviewer-administered. This ensured that those unable to read and write could fully participate and also to ensure optimal capturing of all the needed information. The questionnaire included questions on socio-demographic characteristics (12 questions), utilization of antenatal care services (18 questions). The questionnaire will be first developed in English and then translated later to Kiswahili which is the national language of Tanzania and the language used by the study population. The questionnaire are adopted and modified from a safe motherhood questionnaire developed by the Maternal Neonatal Program of Jhpiego and modified to fit the Tanzanian context [11] ,also from Tanzania Demographic and Health Survey 2015/2016 and from Nepal Demographic and Health Survey [12] .

**Measurements of variables**

**Antenatal care service utilization:**

Adequate antenatal care service utilization will be measured by :

i. Attended by skilled health personnel (provided by a nurse or a doctor)

ii. Timely (initial visit during the first trimester of pregnancy)

iii . Sufficient ( 4 or more visits during pregnancy), iv. Appropriate contents /services (visits included at least 13 out of 15 of the recommended basic care procedures or contents). The sample will be divided into two outcome categories: i) Received adequate antenatal care (delivered by skilled health personnel, timely, sufficient and with appropriate content).ii) received inadequate antenatal care (services which did not fully comply with the above criteria). This method of scoring has been previously used in Mexico to measure the adequacy of antenatal health care. It was adopted and modified to fit the Tanzania content [15].

**Data analysis**

In this study, data will be analyzed by using the Statistical Product for Service Solutions (SPSS) software program version 21. Before conducting the analysis, the error checking (data cleaning) will be performed by using Frequency distribution tables to see if all the data were entered correctly. Each variable will manually be cross-checked to ensure validity and reliability of the findings, frequencies to determine their distributions, means, standard deviations, kurtosis, and skewness. Scores that will be out of range will be corrected to avoid distortion of the statistical analysis. Both descriptive and inferential analyses will be carried out as per the objectives of the study.

Descriptive analysis will be used to analyze participant’s characteristics to determine the frequencies and percentages of their distributions between the two groups. Regression analysis will also be used to establish the association between the Interactive SMS alert system and the outcome variables.

**Discussion .**

In this protocol Interactive messaging alert system (IMAS) is described which aim at improving ANC service utilization. It helps to ensure early diagnosis; treatment and proper follow up. It also empower pregnant women through greater access to health information, and communications tool which may aid self care, shared decision making, and proper follow up for positive pregnant outcomes and preventing undesirable birth outcome by minimizing the effect caused by the first and second delays.

In the literature, there have been many interventions which seek to address the issue of maternal mortality and morbidity. Some interventions have focused on improving birth and emergence preparedness and others have focused on ANC service utilization and knowledge on obstetric and newborn danger signs.

An intervention in South Africa, investigated how an interactive mobile messaging program can promote safe motherhood and improve pregnancy outcomes. The program showed positive results in ANC service utilization and in giving health education during pregnancy [16].

A study done in Rwanda on designing and implementing an innovative SMS-based alert system (Rapid SMS-MCH) to monitor pregnancy and reduce maternal and child deaths between May 2010 and April 2011. Their aim was to monitor pregnancy and reduce bottlenecks in communication associated with maternal and newborn deaths. The intervention reported an increase in usage of health facilities; attendance rate and access to emergency healthcare were all improved through the use of an interactive SMS platform strengthening. In addition, the relationship between the pregnant women and their midwives was improved [17].

Furthermore another intervention study in Zanzibar assessed the effect of SMS on skilled delivery rates and access to emergency healthcare. The women were followed up by midwives 42 days post pregnancy. If any women experienced pain or problems (warning signs) related to the risks that had been delineated by the midwife, the midwife received an SMS from the women explaining their symptoms; the midwife would contact the doctor and ambulance. The effectiveness of this system increased the patients’ confidence and trust in their midwives. Pregnancy complications were reduced by 42% through the utilization of this system, and antenatal healthcare was improved by 91% and institutional deliveries by 51%, respectively [18]

Another intervention going on now in Tanzania is Wazazi Nipendeni (Love me, Parents). Is a national safe motherhood social and behavior change communication (SBCC) campaign, aim is to encourage Tanzanian women and their partners to take steps for a healthy pregnancy and safe delivery led by the Ministry of Health and Social Welfare Reproductive and Child Health Section in coordination with the National Malaria Control Program (NMCP) and the mHealth Tanzania Public Private Partnership.

This provides evidence that if effectively utilized, interventions can improve antenatal services utilization. SMS technology can positively influence health-seeking behavior and satisfaction in low-resource settings. Mobile technologies make people more contactable and as such offer a useful tool to deliver education and improve health-seeking behavior or health-related lifestyle decisions. We believe our study in its current design will result in accurate and relevant findings.

**List of Abbreviations**

**ANC**- Antenatal care

**MMR-** Maternal Mortality Ratio

**MoHCDGEC-** Ministry of Health Community Development Gender, Elderly and Children

**TDHS-**Tanzania Demographic and Health Survey

**WHO-** World Health Organization

**CR**- Complication Readiness

**FANC**- Focused Antenatal Care

**HF**- Health facility

**UDOM**- University of Dodoma

**IBPACR**- Individual Birth Preparedness and Complication Readiness

**SBA**- Skilled Birth Attendant

**IMAS**- Interactive Messaging Alert System

**EMOC**- Emergency Obstetric Care.

**BEMOC**- Basic Emergency Obstetric Care

**NBS**- Nation Bureau of Statistics

**SMS-** Short Message Service

**MDG5**- Millennium Development Goal 5

**SDG3**- Sustainable Development Goal 3

**Declarations**

**Ethics approval and consent to participate**

Ethical clearance will be sought from UDOM, Institutional Research Review committee. All information obtained from each respondent will be identified using an identity number instead of their names on the questionnaire to maintain confidentiality. Human right, privacy, and confidentiality will be considered in this study. Research objectives, Risk, and benefits of the study will be explained well to the participants. Verbal and written consent will be asked of participants and the questionnaire will be answered voluntarily. Also, the control group will not be denied their right to ANC services, instead they will also be followed and continue with the normal standard care being offered in the local clinics. Participants’ names and other identifying information will be held in a computer data base separate from the completed questionnaires.

### Consent for publication

Not applicable.

### Availability of data and materials

Not applicable.

### Competing interests

The authors declare that they have no any competing interests in this study protocol.

### Acknowledgements

Heartfelt thanks to the University of Dodoma, local leaders of Dodoma Municipal and study participants for their willingness to participate and contribute to this study and host research teams during research activities.

**Authors’ contributions**

All authors contributed to the design of the study protocol. TJM wrote the final manuscript, which was reviewed by all other authors. All authors read, contributed to and approved the final manuscript.

**Acknowledgements**

The authors thank the University of Dodoma for providing sponsorship in development of this study protocol and implementation of the protocol.

**References**

1. KGross K, Joanna A, Kessy F, Constanze P. Antenatal care in practice an exploratory study in antenatal care clinics in the Kilombero Valley, south-eastern Tanzania BMC Pregnancy and Childbirth Full Text. BMC Pregnancy and Childbirth; 2011.

2. World Health Organization. WHO Maternal mortality Fact sheet. Geneva: media centre; 2016.

3. Lassi ZS, Mansoor T, Salam RA, Das JK, Bhutta ZA. Essential pre-pregnancy and pregnancy interventions for improved maternal, newborn and child health. Reprod Health. BioMed Central Ltd; 2014;11 Suppl 1(Suppl 1):S2.

4. Ministry of Health Community Development Gender Elderly and Children (MoHCDGEC) [Tanzania, Mainland]. Tanzania Demographic and Health Survey and Malaria Indicator Survey (TDHS-MIS) 2015-16. Dar es Salaam, Tanzania, and Rockville, Maryland, USA; 2016.

5. Shija AE, Msovela J, Mboera LEG. Maternal health in fifty years of Tanzania independence: Challenges and opportunities of reducing maternal mortality. Tanzan J Health Res. 2011;13(5):352–64.

6. Ministry of Health, Community Development, Gender E and C. The National Road Map Strategic Plan To Accelerate Reduction of Maternal , Newborn and Child Deaths in Tanzania. 2015.

7. Alkema L, Chou D, Hogan D, Zhang S, Moller A, Gemmill A, et al. Global , regional , and national levels and trends in maternal mortality between 1990 and 2015 , with scenario-based projections to 2030 : a systematic analysis by the UN Maternal Mortality Estimation Inter-Agency Group. Lancet [Internet]. World Health Organization. Published by Elsevier Ltd/Inc/BV. All rights reserved.; 387(10017):462–74. Available from: http://dx.doi.org/10.1016/S0140-6736(15)00838-7

8. National Bureau of Statistics Ministry of Finance Dar es Salaam, And, Office of Chief Government Statistician Ministry of State, President Office SH and GG. Mortality and Health National. 2015;

9. Ekirapa E, Muhumuza R, Tetui M, George A. Effect of a participatory multisectoral maternal and newborn intervention on maternal health service utilization and newborn care practices a quasi-experimental study in three rural Ugandan districts. Rural Uganda: Global Health action; 2017.

10. Patra P, Size S. Pratap Patra. Sample Size in Clinical Research. 2012;1(1).

11. Jhpiego. birth preparedness. 2004;

12. Division P, Health M, Era N, International ICF. Nepal. 2011;

13. Mpembeni, Rose N M,Japhet Z.kilewo,Melkzedeck T Leshabari,Siriel N Massawe HM. Use pattern of maternal health services and determinants of skilled care during delivery in Southern Tanzania implications for achievement of MDG-5. BMC Pregnancy and Childbirth; 2007.

14. Bintabara D, Mohamed MA, Mghamba J, Wasswa P, Mpembeni RNM. Birth preparedness and complication readiness among recently delivered women in chamwino district , central Tanzania : a cross sectional study. ??? [Internet]. ???; 2015;1–8. Available from: ???

15. Heredia-Pi IA, Servan-Mori E, Darney BG, Reyes-Morales H, Rafael Lozano. Measuring the adequacy of antenatal health care a national cross-sectional study in Mexico. Mexico: World Health Organization (WHO); 2016.

16. Xiong K, Kamunyori J, Sebidi J. The MomConnect helpdesk: How an interactive mobile messaging programme is used by mothers in South Africa. BMJ Glob Heal. 2018;

17. Ngabo F, Nguimfack J, Nwaigwe F, Mugeni C, Muhoza D, Wilson DR, et al. Designing and Implementing an Innovative SMS-based alert system (RapidSMS-MCH) to monitor pregnancy and reduce maternal and child deaths in Rwanda. Pan Afr Med J. 2012;13:31.

18. Lund S, Hemed M, Nielsen BB, Said A, Sadick K, Makungu MH R V. Mobile phones as a health communication tool to improve skilled attendance at delivery in Zanzibar a cluster-randomised controlled trial. Zanzibar: PubMed; 2012.
