# Supplementary material for: The effectiveness of interactive mobile health technologies in improving antenatal care service utilization in Dodoma region, Tanzania: A quasi—Experimental study
Source: PLOS Digit Health. 2023 Aug 16;2(8):e0000321. doi: 10.1371/journal.pdig.0000321 (PMC10431653; doi:10.1371/journal.pdig.0000321)
Supplement: Attachment — Submitted filename: Response to Reviewers.docx [file pdig.0000321.s006.docx]

POINT BY POINT RESPONSE TO EDITOR AND REVIWERS COMMENTS

Dear editor, I would like to appreciate the comments given by you and reviewer on the manuscript submitted as indicated below together with the amendment made:

**Response to editor**

| **SN** | **Comments** | **Response** |
| --- | --- | --- |
| 1 | The recruited sample does not seem to be representative for the population, given that only participants could be enrolled who used ANC services early. Why did the authors decide for this criterion? This limitation should be reflected on in the discussion. | We decided to recruit pregnant women who started their ANC services early so that we could have enough time for follow up to see the effectiveness of the intervention as per World Health Organization standards.  -This has been added as one of the limitation on the discussion section on page 22 |
| 2 | Please Provide the formula used for estimating the required sample size | Thank you for the comment: The formula for sample size calculation as been added as suggested [see Page 8, Line 17] |
| 3 | Please provide more information about the intervention, especially on the intervention messages: What messages were sent? | This has been explained in page 9 with the sub heading ‘**Overview of the Intervention’** [see Page 11. Line 9 – 21] |
| 4 | The discussion describes additional results from a t-test. All results should be provided in the results section only, and be interpreted and discussed in the discussion section. | Thanks for the comment: This was mistakenly included and it has been removed as shown in page 17 |
| **REVIEWER 1** | | |
| 1 | The paper is well written and easy to follow. However, the authors have used the phrase "in due regard" which i think can be replace with "in this regard". Please consult language editor. | Thanks for the comment: The phrase "in due regard" has been replace with"in this regard" throughout the document. |
| 2 | This is where you are stating the aim of the study - (this study assessed efficacy of interactive mobile health technologies ...), this does not much with your title and the rest of the study. I think the right word is effectiveness not efficacy. | The sentence has been revised and amended as suggested by the reviewer [see Page 7, Line 1 – 3] |
| 3 | Methodology, results and discussion: Methodology and results are well explained. | Thanks for your comments |
| 4 | Discussion need to be improved. You have discussed your results based of four references. I think you need to engage more with literature. | Thanks for your comments; The discussion section has been revised and improved as suggested. |
| 5 | Conclusion.  In terms or novelty, this study offers none. Thus, you can bring in recommendations that can inform mobile health designers, implementers and policy makers. Here you need to think out of the box because this is the contribution of the study and it will make the study stronger. | Thanks for the comments; more recommendations have been added as suggested by the reviewers. [See Page 22, Line 8 – 13] |
| **REVIEWER 2** | | |
|  | Thank you for the opportunity to review the paper. While the intervention is potentially interesting, the description of the study, the methods and the analysis should be strengthened. It may be useful to review published papers which conduct bivariate and multivariate analysis to see how they explain some of the different steps. I also think some of the references need to be reviewed. Global recommendations since 2016 recommend 8 antenatal care contacts. I understand Tanzania still recommends four, so reference should be made to the Tanzania national norms. | Thank you for taking your time to review our paper.  In the current version of our paper we addressed all the comments and suggestions you provided throughout the document |
|  | Specific comments |  |
| 1 | it is not clear what that is and it needs to be explained in the background. | Thanks for the comments; The mobile health technologies has been clearly explained in the Methods section under subtitle “Overview of the intervention” together with Fig 1; [see Page 11, Line 6 – 19] |
| 2 | randomly selected is misleading- as the intervention area and control area were determined and from there it is not clear how participants were selected. | Thanks for the comments, although, the intervention area and control were predetermined, but participants were randomly selected |
| 3 | the author summary should not need to convince the reader about the importance of antenatal care but should focus on how mobile technology can be used to increase the uptake of anc. | Thanks for the comments ; The summary has been revised and amended as suggested by the reviewer |
| 4 | please verify the reference - again I am not sure this is the best source for this perhaps the authors can look at the recent estimates https://www.who.int/publications/i/item/9789241516488  which confirms Tanzania to have high rates of MMR | Thanks for the comments; The reference has been confirmed as suggested by the reviewer |
| 5 | Birth preparedness and complications readiness plan | The sentence has been revised and amended as suggested by the reviewer [see Page 5, Line 21] |
| 6 | I am not sure study 9 is pertinent given the availabiltiy of a more recent systematic review - please note, there seems to be a mistake in the reference.  The conclusion says: All studies showed at least some evidence of effectiveness at changing behavior to improve antenatal care attendance, postnatal care attendance, or childhood immunization rates. However, many of the studies were observational and further rigorous evaluation of mHealth programs is needed in a broader variety of settings.  I think you can mention this and still justify your study as contributing to the literature. | The reference section has been updated as suggested by the reviewer  Also, the conclusion has been amended as suggested by the reviewer |

**Reviewers’ comments**
