# Supplementary material for: The effectiveness of interactive mobile health technologies in improving antenatal care service utilization in Dodoma region, Tanzania: A quasi—Experimental study
Source: PLOS Digit Health. 2023 Aug 16;2(8):e0000321. doi: 10.1371/journal.pdig.0000321 (PMC10431653; doi:10.1371/journal.pdig.0000321)
Supplement: Attachment — Submitted filename: Response to reviewers comments.docx [file pdig.0000321.s007.docx]

POINT BY POINT RESPONSE TO EDITOR AND REVIWERS COMMENTS

Dear editor, I would like to appreciate the comments given by you and reviewer regarding our manuscript. We have addressed all your comments and those highlighted by the reviewer so that to make our manuscript looks good for possible publication.

Below are line by line comments and response made in the main document

**Response to editor and reviewer**

| SN | Comments | Response |
| --- | --- | --- |
| 1 | Please describe the intervention in more detail. For instance, what tips did women receive | - The description of the intervention including the health education messages that were sent to pregnant women has been added as shown in page 11-12 of the manuscript |
| 2 | Please extend the discussions by additional references to previous research - it still includes only a very small number of references and so seems to ignore important parts of the literature | - The discussion section has been extended by adding more references from previous studies as shown in page 21-22 |
| 3 | **Reviewer #1:**  Please use the language editor to proofread the manuscript. Check page 11 line 16 -19. Line 16 the sentence is not complete. And the next sentence is too long | - Thanks for the comments, the sentences have been revised and amended as suggested by the reviewer. Please see Page 11, Line 16 – 23. |
